# Gut microbiome changes in mouse, Mongolian gerbil, and hamster models following *Clostridioides difficile* challenge

**DOI:** 10.3389/fmicb.2024.1368194

**Published:** 2024-04-04

**Authors:** Shuangshuang Wan, Peijun You, Qikai Shi, Hui Hu, Lu Zhang, Leyang Chen, Ziyi Wu, Shan Lin, Xiaojun Song, Yongneng Luo, Yaxuan Wang, Feng Ju, Dazhi Jin, Yu Chen

**Affiliations:** ^1^School of Laboratory Medicine, Hangzhou Medical College, Hangzhou, China; ^2^Key Laboratory of Biomarkers and In Vitro Diagnosis Translation of Zhejiang Province, Hangzhou, China; ^3^Environmental Microbiome and Biotechnology Laboratory, School of Engineering, Westlake University, Hangzhou, China; ^4^School of Clinical Medicine, Hangzhou Medical College, Hangzhou, China; ^5^TEDA Institute of Biological Sciences and Biotechnology, Nankai University, Tianjin, China; ^6^Laboratory Medicine Center, Department of Clinical Laboratory, Zhejiang Provincial People’s Hospital, Hangzhou Medical College, Hangzhou, China; ^7^National Center for Clinical Laboratories, Institute of Geriatric Medicine, Chinese Academy of Medical Sciences, Beijing Hospital/National Center of Gerontology, Beijing, China

**Keywords:** *Clostridioides difficile*, rodent models, Mongolian gerbils, gut microbiome, *Lactobacillaceae*, *Akkermansia*

## Abstract

**Introduction:**

*Clostridioides difficile* infection (CDI), as well as its etiology and pathogenesis, have been extensively investigated. However, the absence of suitable CDI animal models that reflect CDI symptoms and the associated gut microbiome changes in humans has limited research progress in this field. Thus, we aimed to investigate whether Mongolian gerbils, which present a range of human pathological conditions, can been used in studies on CDI. Methods: In this study, we infected Mongolian gerbils and two existing CDI model animals, mice and hamsters, with the hypervirulent ribotype 027 C. difficile strain, and comparatively analyzed changes in their gut microbiome composition via 16S rRNA gene sequencing.

**Methods:**

In this study, we infected Mongolian gerbils and two existing CDI model animals, mice and hamsters, with the hypervirulent ribotype 027 C. *difficile* strain, and comparatively analyzed changes in their gut microbiome composition via 16S rRNA gene sequencing.

**Results:**

The results obtained showed that C. *difficile* colonized the gastrointestinal tracts of the three rodents, and after the C. *difficile* challenge, C57BL/6J mice did not manifest CDI symptoms and their intestines showed no significant pathological changes. However, the hamsters showed explosive intestinal bleeding and inflammation and the Mongolian gerbils presented diarrhea as well as increased infiltration of inflammatory cells, mucus secretion, and epithelial cell shedding in their intestinal tissue. Further, intestinal microbiome analysis revealed significant differences with respect to intestinal flora abundance and diversity. Specifically, after C. *difficile* challenge, the Firmicutes/Bacteroidetes ratio decreased for C57BL/6J mice, but increased significantly for Mongolian gerbils and hamsters. Furthermore, the abundance of Proteobacteria increased in all three models, especially in hamsters, while that of Verrucomicrobia only increased significantly in C57BL/6J mice and Mongolian gerbils. Our results also indicated that differences in the relative abundances of *Lactobacillaceae* and *Akkermansia* were primarily responsible for the observed differences in response to C. difficile challenge.

**Conclusion:**

Based on the observed responses to C. *difficile* challenge, we concluded for the first time that the Mongolian gerbil could be used as an animal model for CDI. Additionally, the taxa identified in this study may be used as biomarkers for further studies on CDI and to improve understanding regarding changes in gut microbiome in CDI-related diseases.

## 1 Introduction

*Clostridioides difficile* is a gram-positive spore-forming obligate anaerobic bacterium. In patients with long-term use of antibiotics or low immunity, the balance of intestinal flora is disrupted, such that *C. difficile* adheres to host intestinal epithelial cells, rapidly proliferates the intestinal niche, and produces toxins, such as *C. difficile* toxin A [TcdA] and *C. difficile* toxin B [TcdB] as well as binary toxins. These toxins trigger a cascade of cellular events that lead to fluid secretion, inflammation, and tissue damage, which bring about the symptoms of *C. difficile* infection (CDI), such as pseudomembranous colitis, toxic megacolon, and even death (Juul et al., 2018; Zhou et al., 2019; Rao and Malani, 2020).

CDI and its etiology and pathogenesis have been extensively investigated (Wydau-Dematteis et al., 2018; Castro-Córdova et al., 2021; Luo et al., 2022). Specifically, the progression of CDI has been studied using various animal species, including hamsters, guinea pigs, rabbits, mice, and rats. Among these animals, hamsters have been most widely used in CDI research (Borriello et al., 1987; Best et al., 2012). However, they often develop fulminant cecitis and ultimately die within 1–3 days following challenge with *C. difficile*. This phenomenon is rarely observed in the clinical course of CDI in humans, in whom CDI shows a more gradual onset, with death being rare (Abutaleb and Seleem, 2021; Singh et al., 2022). Furthermore, a mouse model of antibiotic-induced *C. difficile-*associated colitis that presents CDI symptoms similar to those in humans, including diarrhea, pseudomembrane formation, variable disease severity, and recovery of disease after vancomycin therapy, has been established. Thus, it serves as an important tool for exploring CDI pathogenesis (Wu et al., 2022). However, mouse models do not always show overt clinical signs of CDI, despite remaining colonized with *C. difficile* (Reeves et al., 2011). The Mongolian gerbil (*Meriones unguiculatus*) is an efficient and cost-effective rodent that shows several features which are absent in mice or rats, including relatively low intragastric pH, sensory specializations, and social patterns, similar to humans (Sheh and Fox, 2013; Zorio et al., 2019). Their use in the study of several pathogenic diseases is also very common. For example, studies involving the use of Mongolian gerbils as a model of infection have shown that *Helicobacter pylori*-infected Mongolian gerbils develop high levels of gastric inflammation and often present with gastric adenocarcinoma or ulceration (Beckett et al., 2018). In contrast, *H. pylori* can only induce mild inflammation in many mouse models, and it is difficult in establishing infection in mice (Kabir et al., 1997; Lee et al., 1997). However, it is still unclear whether Mongolian gerbils can be used as a novel model. Gut microbes modulate nutrition and immune function and are associated with a growing number of health and disease states (Sharon et al., 2014; Lynch and Pedersen, 2016). For example, in the healthy state, the structure and diversity of the human gut microbiome is relatively stable (Lloyd-Price et al., 2017). However, in the diseased state, this balance is significantly dysregulated. In recent decades, changes in gut microbiome structure have been increasingly recognized as holding promise with respect to advancing CDI research. Antibiotic exposure leading to microbiome disruption and overgrowth of *C. difficile* is a major risk factor for the development of CDI (Vakili et al., 2020a). It has also been demonstrated that CDI is associated with severe gut microbiome disruption (Buffie et al., 2012; Zhang et al., 2022). CDI-related structural and functional changes in gut microbiome have been investigated in human, and there were significant differences on the composition of gut microbiome between patients with CDI and healthy persons (Berkell et al., 2021). Thus, it was reasonable that change in gut microbiome was recognized as a biomarker to predict development of CDI. Moreover, structural changes in gut microbiome have also been used to comparatively analyze the features for CDI between rodents and human, indicating that CDI similarly led to microbiome dysbiosis of animal (Gu et al., 2020). Nevertheless, it has been still unclear whether changes in gut microbiome configuration were associated with diverse outcomes in different animals after *C. difficile* challenge. Therefore, in this study, we experimented with three rodent-based CDI models (mice, hamsters, and Mongolian gerbils) and 16S rRNA gene sequencing and analyzed changes in their gut microbiome structure, owing to *C. difficile* challenge.

## 2 Materials and methods

### 2.1 Bacterial strain and growth conditions

*Clostridioides difficile* strain 4118, obtained from American Type Culture Collection (www.atcc.org), was cultured anaerobically at 37°C on brain heart infusion agar (Oxoid Ltd., Basingstoke, UK) supplemented with 1.5% (w/v) glucose, 0.1% (w/v) L-cysteine, 1.5% (w/v) agar, and 0.1% (w/v) sodium taurocholate. Thereafter, the strain was inoculated into 500 mL of tryptone yeast broth containing 3% tryptone, 2% yeast extract, and 0.1% sodium thioglycolate and further cultured anaerobically at 37°C for 7 days, after which spores were harvested via centrifugation performed at 1,500 *g* and 4°C for 20 min, washed six times with ddH_2_O, and resuspended in phosphate buffered saline (PBS). Next, heat-shocking was performed at 65°C for 30 min to remove bacteria. The spores were then 10-fold diluted and anaerobically cultured at 37°C for 48 h after, which the number of spores was calculated. *C. difficile* was isolated from stool samples from the control and infected rodents, and four toxin genes (*tcdA*, *tcdB*, *cdtA*, and *cdtB*) were identified as previously reported (Indra et al., 2008; Griffiths et al., 2010).

### 2.2 Animals

Male C57BL/6 J mice (age, 6–8 weeks) were purchased from Shanghai Laboratory Animal Co., Ltd. (Shanghai, China). Male golden hamsters (age, 6–8 weeks) were purchased from Beijing Weitong Lihua Laboratory Animal Technology Co., Ltd. (Beijing, China), and male Mongolian gerbils (age, 10–12 weeks) were purchased from the Experimental Animal Center of Hangzhou Medical College, China. The animals were housed at the Laboratory Animal Center at Hangzhou Medical College (23°C ± 2°C room temperature, 55% ± 5% relative humidity, and a 12 L:12D photoperiod), at a density of three animals per cage in a specific pathogen-free animal facility. The experimental protocols were approved by the Animal Care and Use Committee of the abovementioned institution (approval number SYXK 2017-0013). Furthermore, all the animal experiments were performed in accordance with Animal Research: Reporting of *In Vivo* Experiments guidelines (Percie Du Sert et al., 2020). The mice were euthanized 5 days after anesthesia administration via the intraperitoneal injection of chloral hydrate (375 mg/kg of body weight).

### 2.3 Induction of *Clostridioides difficile* challenge and monitoring of animals

All the animals (mice, hamsters, and Mongolian gerbils) were randomized into groups with corresponding average body weights to establish the necessary durations of antibiotic pretreatment. The control and challenge groups for each species comprised five animals. Furthermore, the fresh antibiotic mixture, which comprised kanamycin (0.4 mg/mL), gentamicin (0.035 mg/mL), metronidazole (0.215 mg/mL), colistin (850 U/mL), and vancomycin (0.045 mg/mL), was purchased from Sigma–Aldrich (St. Louis, MO, United States). Once this mixture was prepared, it was added to the drinking water of the animals, which was replaced daily for 3 days. After this 3-day antibiotic pretreatment, the animals were further treated with clindamycin (10 mg/kg) for 1 day, as described previously (Chen et al., 2008) and then challenged with 5 × 10^4^ colony-forming units of *C. difficile* strain 4,118 via oral gavage. The animals were then monitored for signs of disease, such as diarrhea, wet tail, bow back, malaise, and weight loss. After 5 days, the animals were euthanized, and their stool samples and intestinal tissues were collected for analysis.

### 2.4 Detection of TcdB toxins in stool samples via ELISA

TcdB toxins in stool samples from the control and infected rodents were detected via ELISA. The collected stool samples were suspended in 10% (wt/vol) PBS (pH 7.4). Thereafter, the fecal suspensions were seeded into 96-well plates and incubated at 4°C overnight. This was followed by washing with PBST (PBS pH 7.4 + 0.05% tween-20) after which the plate was blocked with 0.5% BSA at 37°C for 2 h. TcdB polyclonal antibodies (List Biological Laboratories Campbell, CA, United States; Chicken IgY, 1:1000 dilution ratio) were used for the first hybridization process, while goat anti-chicken IgY secondary antibody and horse radish peroxidase (Invitrogen, Carlsbad, CA, USA; 1:5000 dilution ratio) were used for the second hybridization. The ELISA-TMB Chromogenic Reagent kit (Sangon, Shanghai, China) was used to determine the absorbance at 450 nm. Pure TcdB toxin (List Biological Laboratories) was used as the positive control, and standard curves were generated.

### 2.5 Histopathological analysis

Resected ileal loops were fixed in 4% formaldehyde, buffered with PBS, and embedded in paraffin. Next, the tissue sections were deparaffinized, stained with hematoxylin and eosin, and subjected to histological analysis. Tissue injuries were scored by two blinded pathologists, who independently graded inflammation according to five grades based on the degrees of chronic inflammation and tissue damage, as described previously (Rugge et al., 2021).

### 2.6 Extraction and amplification of genomic DNA

Total genomic DNA was extracted from 0.25 g of intestinal contents using the Dneasy^®^ PowerSoil^®^ Pro Kit (QIAGEN, Hilden, Germany), according to the manufacturer’s protocol. DNA concentration and purity were verified via electrophoresis using 1% agarose gel. Furthermore, the genomic DNA was diluted to 1 ng/Μl using sterile water, and the 16S V3–V4 region of the 16S rRNA gene was amplified using specific primers (341F-5′-CCT AYG GGR BGC ASC AG-3′ and 805R-5′-GAC TAC HVG GGT ATC TAA TCC-3′). All the PCR assays were performed using 15 μL of PCR Master Mix (New England Biolabs, Ipswich, MA, United States), 2 μM forward and reverse primers, and approximately 15 ng of template DNA. The PCR program was as follows: denaturation at 94°C for 3 min, 5 cycles of denaturation at 94°C for 30 s, annealing at 45°C for 20 s, elongation at 65°C for 30 s, 20 cycles of denaturation at 94°C for 20 s, annealing at 55°C for 20 s, elongation at 72°C for 30 s, and 72°C for 5 min. The PCR products obtained were purified using the QIAGEN Gel Extraction Kit (QIAGEN).

### 2.7 16S rRNA gene sequence analysis

The TruSeq DNA PCR Free sample preparation kit (Illumina, San Diego, CA, United States) was used to generate the sequencing library, and an index code was added to acquire operational taxonomic unit (OTU) clusters (sequences with ≥97% similarity were assigned to the same OTU). Taxonomic assignment in terms of 16S rRNA was performed based on the SILVA 132 reference database^1^ using UCLUST^2^ at a 90% threshold. Furthermore, taxonomic assignment based on the ribosomal internal transcribed spacer region was performed using the UNITE reference database (https://unite.ut.ee/, version 01.12.2017) in BLAST at an e-value threshold of 0.001. Furthermore, the determination of phylogenetic relationships based on OTUs, differences in dominant species between different samples (groups), and multiple sequence alignments were carried out using PyNAST software version 1.2 (University of Colorado, Boulder, CO, United States).

### 2.8 Microbial community diversity and functional analysis

Mothur^3^ was used to determine parameters related to the complexity and diversity of the intestinal microbiota of the animals, such as abundance-based coverage estimators (ACE) and Chao 1 richness. Simpson, Shannon, and beta diversity indices on both weighted and unweighted UniFrac were also calculated using QIIME software. Cluster analysis was performed using principal component analysis, principal coordinate analysis, and non-metric multidimensional scaling (NMDS). Furthermore, linear discriminant analysis effect size (LefSe) was performed using LefSe software, and the filter value for the linear discriminant analysis (LDA) score was set to 2. Furthermore, to investigate the functions and pathways involved with *C. difficile* challenge in the test animals, Kyoto Encyclopedia of Genes and Genomes (KEGG) functions were predicted using Tax4Fun software,^4^ and intergroup differences were analyzed in terms of functional abundance differences among the samples.

### 2.9 Statistical analysis

All data were presented as the mean ± SEM and were compared by performing *t*-test using GraphPad Prism software version 8 (San Diego, CA, United States). *p* < 0.05 was considered statistically significant. Furthermore, *p*-values relative to the control group (*p < 0.05, **p < 0.01, ***p < 0.001, and ****p < 0.0001) were determined via one-way analysis of variance (ANOVA).

## 3 Results

### 3.1 Construction of three rodent models with *Clostridioides difficile* challenge

**Figure 1:**
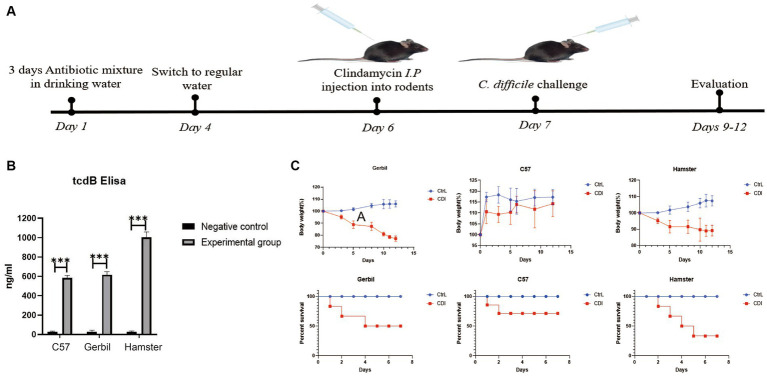
Evaluation of three rodent models with *C. difficile* challenge. **(A)** Schematic representation of animal models with *C. difficile* challenge. **(B)** TcdB content of stool samples from three rodents determined via ELISA. **(C)** Changes in body weight and survival proportions after *C. difficile* challenge. C57, C57Bl/6 J mouse; gerbil, Mongolian gerbil.

Three rodent models with *C. difficile* challenge were established using the classical intragastric administration method, as shown in Figure 1A. Before challenge, the *C. difficile* 4,118 strains with four toxin genes were not detected in stool samples of three rodents (Supplementary Figure S1A). On the second day after intragastric *C. difficile* administration, the stool samples were collected and cultured for *C. difficile*. The results showed that *C. difficile* 4,118 strains were obtained from stool samples of three rodents, and four toxin genes were positive (Supplementary Figure S1B). TcdB was also detected in all stool samples (Figure 1B). This observation indicated that *C. difficile* successfully existed in the intestines of the three rodents and produced the corresponding virulence factors. Based on changes in the body weights of the three rodents and their survival proportions, we found that both Mongolian gerbils and hamsters were susceptible to *C. difficile,* which showed weight loss (Figure 1C). Specifically, all the Mongolian gerbils challenged with *C. difficile* exhibited diarrhea (Supplementary Figure S2D). Furthermore, the survival rate of the hamsters was significantly lower than those of the gerbils and mice (Figure 1C). Our results also indicated that no diarrhea in mice challenged with *C. difficile* (Supplementary Figure S2B), and their weights did not change significantly relative to the weights of the controls (Figure 1C).

### 3.2 Intestinal histopathology of the three model animals after *Clostridioides difficile* challenge

**Figure 2:**
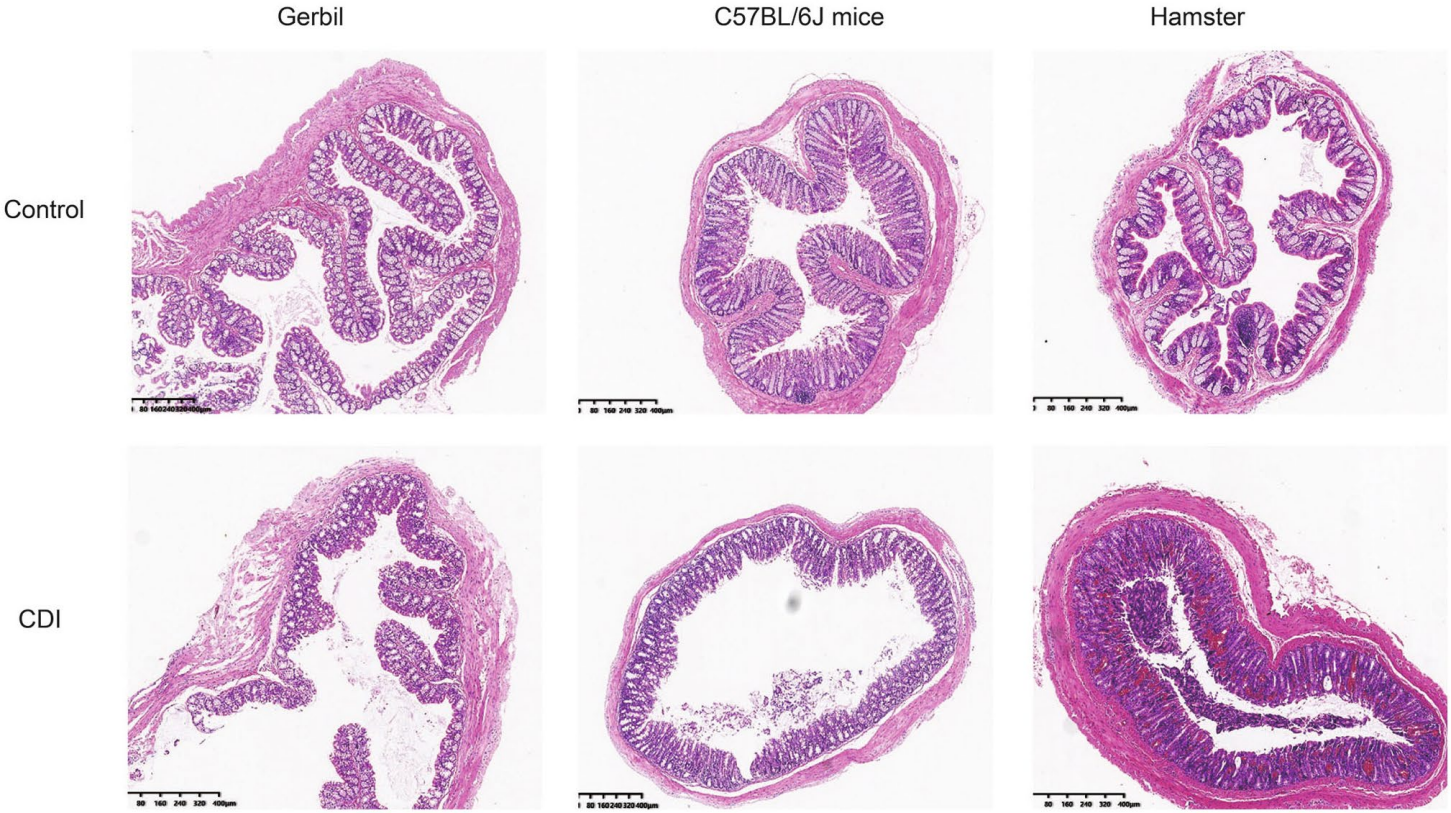
Intestinal histopathology after *C. difficile* challenge in three rodent models. Histopathologic examination of the colon of Mongolian gerbils, mice, and hamsters after *C. difficile* challenge; Gerbil, Mongolian gerbil.

Intestinal histopathology changes were detected in all the rodents (Figure 2). The intestinal mucosal cells of the animals in the three control groups were closely packed and were clearly structured, without hyperemia, edema, and inflammatory infiltration. However, after infection, the hamsters exhibited pathological changes, including explosive intestinal bleeding, and quickly died owing to severe enterocolitis. C57BL/6J mice did not manifest diarrhea. Furthermore, the pathological changes in their intestine were not significant; infection only resulted in the irregular arrangement of myometrium cells, with loose cell arrangement and limited inflammatory cell infiltration. Mongolian gerbils exhibited characteristics that increased infiltration of inflammatory cells into intestinal tissues, including neutrophils, increased mucus secretion and significant epithelial cell shedding. This observation suggested that histopathology in Mongolian gerbil is a typical histologic feature of CDI.

### 3.3 Comparative analysis of intestinal microbial diversity in the three rodent models before and after *Clostridioides difficile* challenge

**Figure 3:**
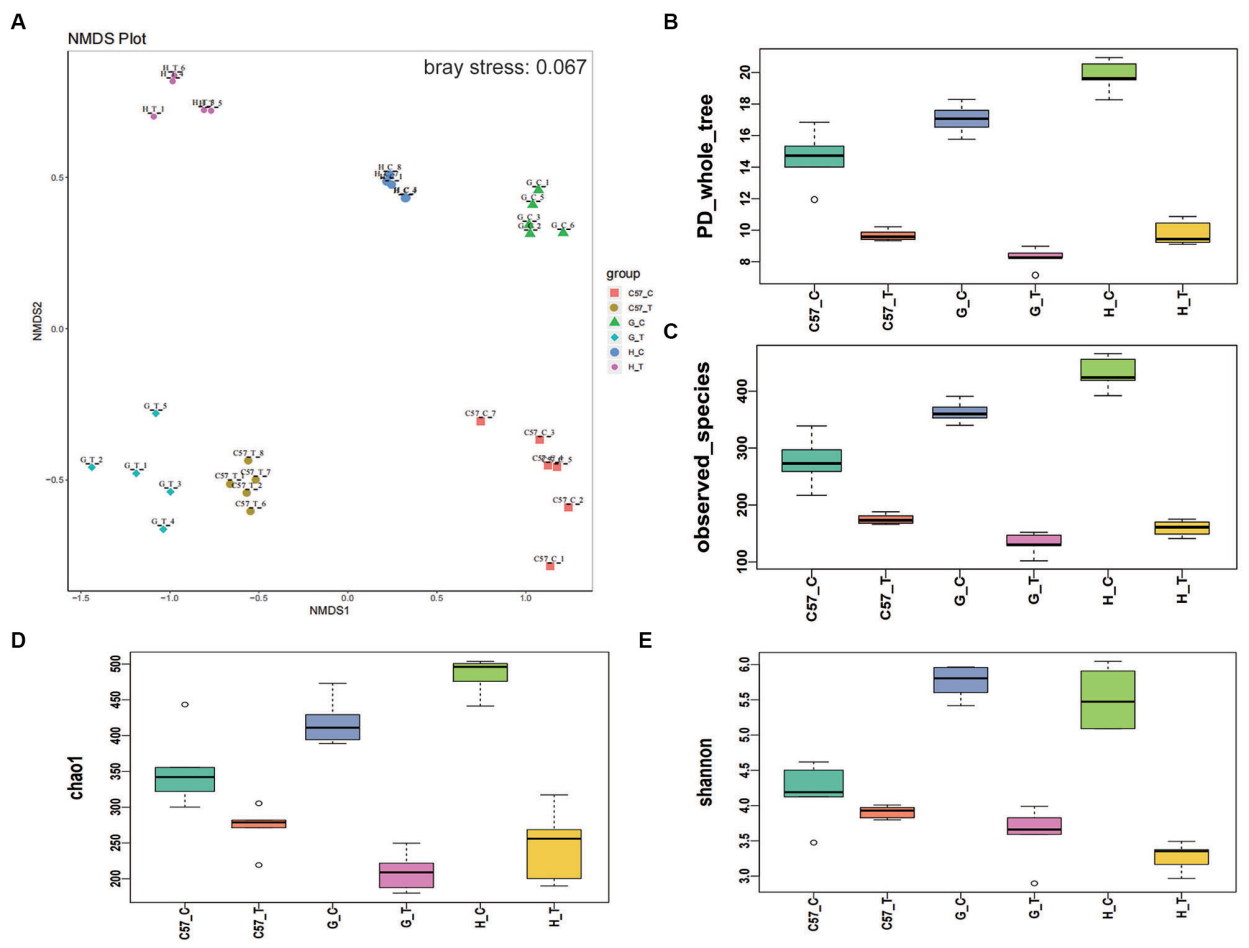
Differences in alpha- and beta-diversity indices between rodent groups after *C. difficile* challenge. Boxplots of group differences between alpha-diversity and beta-diversity indices: **(A)** NMDS, **(B)** phylogenetic diversity whole tree, **(C)** observed species, **(D)** Chao1, and **(E)** Shannon. C, control; C57, C57Bl/6 J mouse; G, Mongolian gerbil; H, hamster; T, treated (i.e., challenged with *C. difficile*), NMDS, non-metric multidimensional scaling.

Thirty intestinal contents were collected from six rodent groups (five CDI-treated rodents and five control rodents for each species) and subjected to microbiota community analysis using 16S rRNA amplicon sequencing. After quality control, 2,020,374 sequences (40,146 reads per sample) were included for downstream analysis. The overall beta-diversity of gut microbiome signatures in the three challenged and three control rodent groups was analyzed via NMDS. The results revealed different community structures for the gut microbiome in each of the six groups. Samples with high community structure similarities were clustered together, whereas samples with large community structure differences were separated. The NMDS results further showed a separation between the three rodent CDI-treated and control groups and, notably, revealed that the least distance existed between the gut microbiomes of the challenged mouse and gerbil (Figure 3A). This finding suggests that the gut microbiome of mice and Mongolian gerbils possibly became similar following CDI. Furthermore, alpha-diversity indices showed higher phylogenetic diversity in the challenged groups than their corresponding control groups. Notably, hamster gut microbiome showed the greatest changes following *C. difficile* challenge, Mongolian gerbils, and lastly mice (Figure 3B). The Chao1 and Shannon indices obtained were consistent with the phylogenetic diversity results (Figures 3C–E).

### 3.4 Analysis of microbial community composition differences among the three rodent CDI models before and after *Clostridioides difficile* challenge

**Figure 4:**
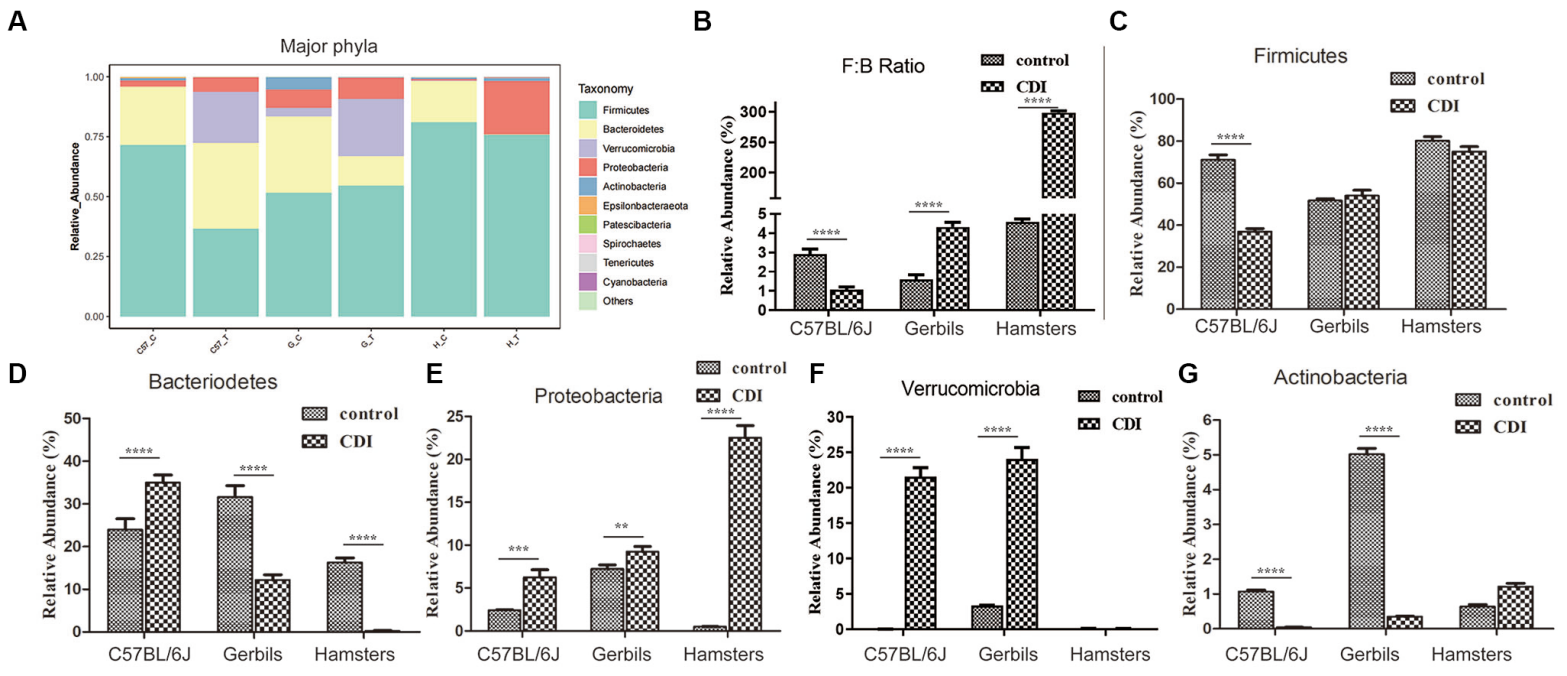
Differences in the relative abundances of major gut bacterial phyla after *C. difficile* challenge. **(A)** Relative abundances of the major phyla in each sample. **(B–G)** Box-plots representing the mean relative abundances of the Firmicutes and Bacteriodetes ratio, Firmicutes, Bacteriodetes, Proteobacteria, Verrucomicrobia, and Actinobacteria in each sample, respectively. *p*-values were determined via ANOVA versus control conditions (**p < 0.01, ***p < 0.001, and ****p < 0.0001); Gerbils, Mongolian gerbil.

To further analyze the relationship between intestinal flora and CDI, we comparatively explored the dynamic alterations in the composition of the intestinal microbiome in the C57BL/6 J mouse, hamster, and Mongolian gerbil models before and after challenge with *C. difficile*. At the phylum level, we observed seven bacterial phyla, of which the four most abundant were Firmicutes, Bacteroidetes, Proteobacteria, and Verrucomicrobia, which jointly contributed to an average sequence ratio of approximately 90%. Actinobacteria, Epsilonbacteraeota, Patescibacteria, and Tenericutes only accounted for approximately 6% of the sequences (Figure 4). The gut microbiomes in the three rodent models appeared to be dominated by Firmicutes, followed by Bacteriodetes, while the relative abundances of Proteobacteria, Verrucomicrobia, and Actinobacteria were very low, ranging from 0.03 to 7.8%. Furthermore, after challenge with *C. difficile*, the relative abundance of Firmicutes in C57BL/6 J mice decreased significantly but was almost unchanged in Mongolian gerbils and hamsters. We also noted that after challenge with *C. difficile*, the relative abundance of Bacteriodetes in the C57BL/6J mice increased but decreased in the two other species, especially in hamsters, in which Bacteroidetes were hardly detected. The Firmicutes to Bacteriodetes ratio also decreased in C57BL/6 J mice but increased in the other two rodent models, especially in hamsters. Furthermore, the three rodent models, especially hamsters, showed an increase in the relative abundance of Proteobacteria, while the relative abundance of Verrucomicrobia showed a significant increase in C57BL/6 J mice and Mongolian gerbils but was very low in hamsters (undetectable). The relative abundance of Actinobacteria in C57BL/6 J mice and gerbils increased but basically remained unchanged in hamsters.

**Figure 5:**
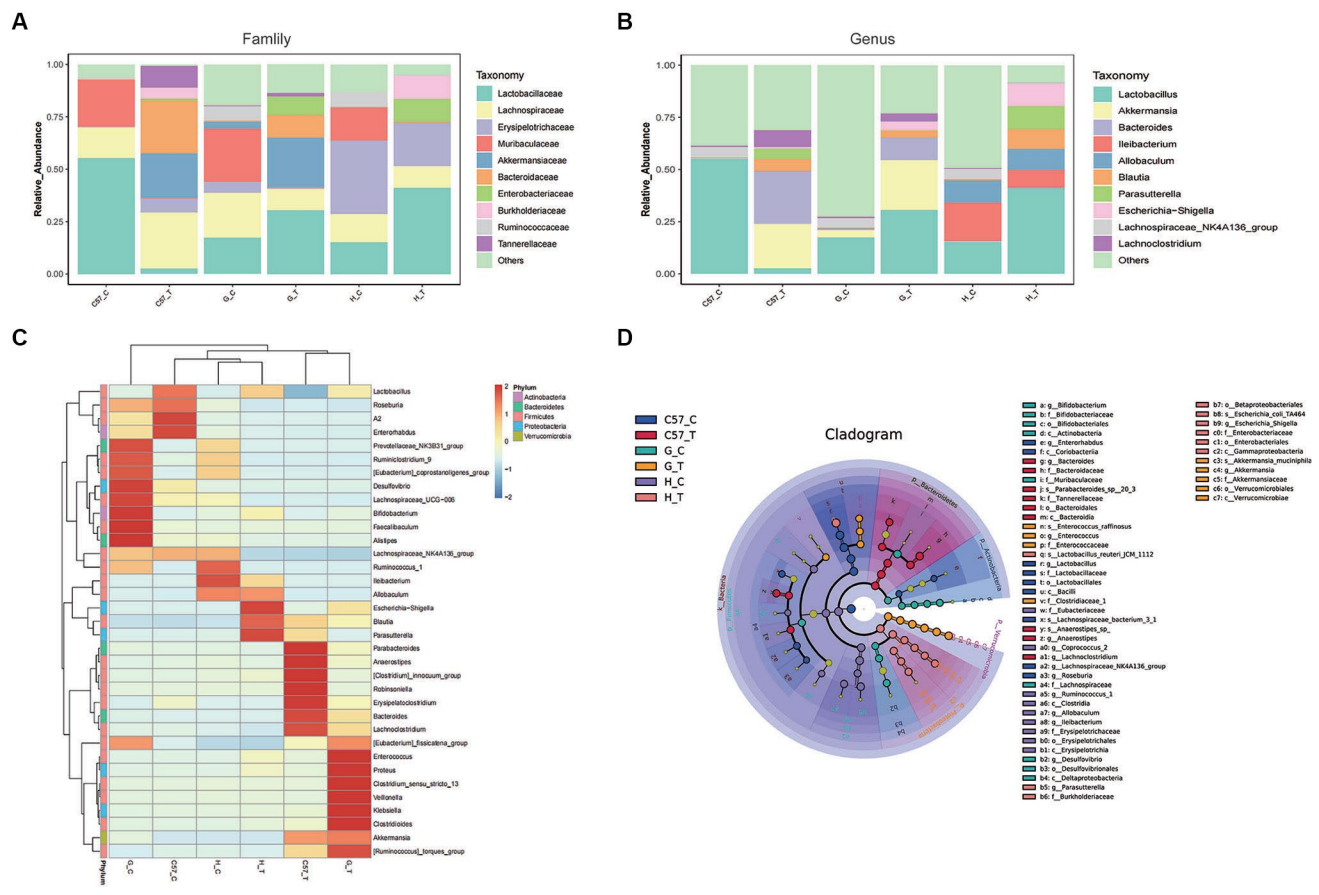
Relative abundance of major gut bacterial families and genera after *C. difficile* challenge in three rodent models. **(A)** Bar graphs representing the relative abundances of major families. **(B)** Bar graphs representing the relative abundances of major genera. **(C)** Heat-map illustrating the hierarchical clustering of operational taxonomic units (OTUs) in each sample. **(D)** LEfSe cladogram representing unique bacterial signatures identified in each sample; the corresponding value of the middle heat map represents the *Z*-value obtained based on the standardized relative abundance of the species in each line; C, control; C57, C57Bl/6 J mouse; G, Mongolian gerbil; H, hamster; T, treated (i.e., challenged with *C. difficile*).

Consistent with phylum-level data, analyses at the family and genus levels showed that the relative abundances of family and genus varied greatly between the different host species and their controls following CDI. For instance, prior to challenge with *C. difficile*, Firmicutes in C57BL/6 J mice appeared to be dominated by the family *Lactobacillaceae* (and the genus *Lactobacillus*) followed by *Lachnospiraceae* (and the genus *NK4A136_group*) (Figures 5A,B). Furthermore, in Mongolian gerbils, Firmicutes predominantly comprised *Lachnospiraceae* (and the genus *NK4A136_group*) and *Lactobacillaceae* (and the genus *Lactobacillus*), followed by *Ruminococcaceae* and *Erysipelotrichaceae*, whereas in hamsters, Firmicutes predominantly comprised *Erysipelotrichaceae* (and the genera *Ileibacterium* and *Allobaculum*) followed by *Lachnospiraceae* (and the genus *NK4A136_group*), *Lactobacillaceae* (and the genus *Lactobacillus*), and *Ruminococcaceae* (and the genus *Blautia*). In the three rodent control groups, Bacteriodetes predominantly comprised the family *Muribaculaceae*. However, the detection rates for families or genera belonging to Proteobacteria or Verrucomicrobia in the three control groups were very low. After challenge with *C. difficile*, Firmicutes in C57BL/6J mice predominantly comprised OTUs belonging to the family *Lachnospiraceae*, followed by *Erysipelotrichaceae*. Notably, compared with the control C57BL/6 J mice, the relative abundances of the genera *Blautia* and *Lachnoclostridium* were higher in challenged mice, while those of the genera *Lactobacillus* and *NK4A136_group* were significantly lower. In Mongolian gerbils, the family *Lactobacillaceae* was the most abundant among Firmicutes. Compared with the corresponding control gerbil group, the relative abundances of genera such as *Lactobacillus, Blautia*, and *Lachnoclostridium* were higher, while that of the genus *NK4A136_group* was very low (undetectable). In hamsters, Firmicutes predominantly comprised OTUs belonging to the family *Lactobacillaceae*, followed by *Erysipelotrichaceae* and *Lachnospiraceae*. Similar to the results obtained for gerbils, compared with the corresponding controls, the genera *Lactobacillus* and *Blautia* were more abundant in challenged Mongolian gerbils, while the genus *NK4A*hamsters*136_group* was undetectable. In contrast, the family *Erysipelotrichaceae* and genera *Ileibacterium* and *Allobaculum* were highly abundant in challenged gerbils. Bacteroidetes in C57BL/6J mice and Mongolian gerbils after *C. difficile* challenge predominantly comprised the family *Bacteroidaceae* (and the genus *Bacteroides*), followed by *Tannerellaceae*. The family *Muribaculaceae* was undetectable in all three models after *C. difficile* challenge. Notably, the relative abundances of the families *Bacteroidaceae* (and the genus *Bacteroides*) and *Tannerellaceae* were higher in C57BL/6 J mice than in Mongolian gerbils.

After challenge with *C. difficile*, *Akkermansia* (a major member of the family *Verrucomicrobiaceae*) showed a high relative abundance in C57BL/6 J mice and Mongolian gerbils but was rarely detected in hamsters (Figures 5A,B). Additionally, the three models challenged with *C. difficile* predominantly comprised the families *Burkholderiaceae* (and the genus *Parasutterella*) and *Enterobacteriaceae* (and the genus *Escherichia−Shigella*), and the relative abundances of *Parasutterella* and *Escherichia−Shigella* were higher in hamsters than in C57BL/6 J mice and Mongolian gerbils, respectively.

The analysis of the top 35 OTUs detected in the three models via heat map clustering before and after challenge with *C. difficile* showed similar patterns as reported above. Notably, after challenge with *C. difficile*, C57BL/6 J, clusters predominantly comprising *Blautia, Erysipelatoclostridium, Anaerostipes, Robinsoniella, Lachnoclostridium, Bacteroides, Akkermansia, Parabacteroides,* and *Parasutterella* were observed in C57BL/6J mice (Figure 5C). In Mongolian gerbils, the clusters predominantly comprised *Blautia, Erysipelatoclostridium, Anaerostipes, Lachnoclostridium, Bacteroides, Akkermansia, Lactobacillus*, *Clostridioides, Klebsiella, Escherichia−Shigella, Enterococcus,* and *Proteus*, and in hamsters, they predominantly comprised *Lactobacillus*, *Ileibacterium, Allobaculum, Blautia, Parasutterella, Escherichia−Shigella, Enterococcus,* and *Proteus*. These features were further confirmed via LEfSe analysis of the major OTUs detected in these groups (Figure 5D).

### 3.5 KEGG analysis of the metabolic functions of the intestinal flora in the three rodent models after *Clostridioides difficile* challenge

**Figure 6:**
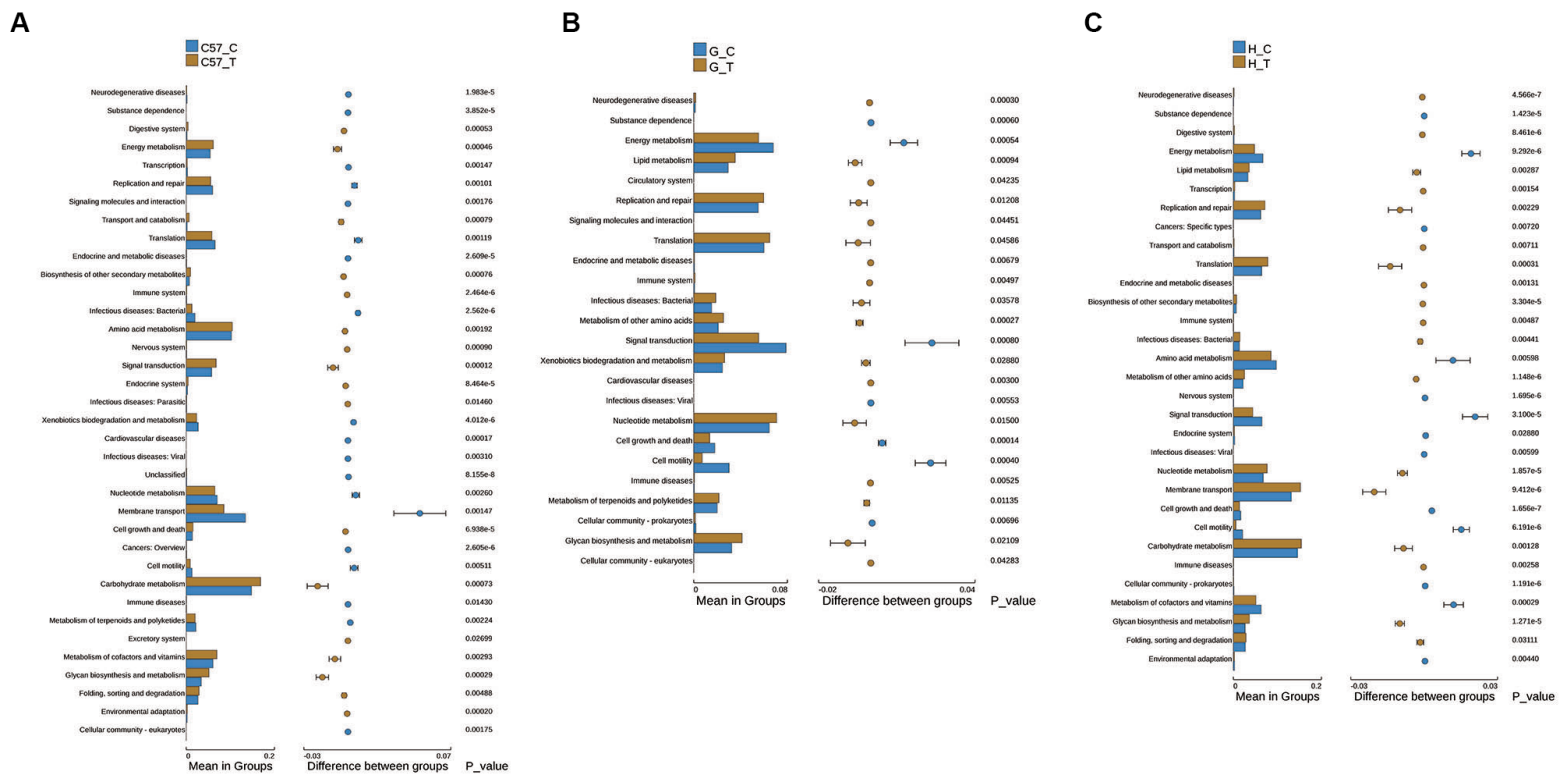
Differential metabolic pathways in functional level 2 determined via KEGG pathway enrichment analysis of the intestinal flora of three rodent models after *C. difficile* challenge. **(A)** C57BL/6 J mice, **(B)** Mongolian gerbils, and **(C)** Hamsters. The right panel shows the difference in confidence between the groups, with the leftmost and rightmost ends of each circle indicating the lower and upper limits of the 95% confidence interval for the mean difference, respectively. The groups represented using individual-colored circles are those with a high mean. The far-right end of the circle represents the intergroup significance test *p*-values for the corresponding differential function; C, control; C57, C57Bl/6 J mouse; G, Mongolian gerbil; H, hamster; T, treated (i.e., challenged with *C. difficile*).

KEGG pathway enrichment analysis showed the involvement of multiple metabolic pathways in *C. difficile* challenge in the three rodent models. Statistically significant differences were also observed among the three models. Difference analysis at the second classification level performed under the conditions: relative abundance >0.1% of the total observed pathways and threshold *p*-value = 0.05. The results showed 36, 24, and 31 differential pathways in C57BL/6 J mice, Mongolian gerbils, and hamsters, respectively (Figure 6). Among the three groups, the terms “amino acid metabolism,” “membrane transport,” “metabolism of cofactors and vitamins,” “signal transduction,” “nucleotide metabolism,” “energy metabolism,” and “glycan biosynthesis and metabolism” dominated gut microbiome functions. Notably, upregulation of “glycan biosynthesis and metabolism” was observed in the three groups, while the other pathways were only consistent for Mongolian gerbils and hamsters but not for C57BL/6 J mice. Additionally, in C57BL/6 J mice, “amino acid metabolism,” “metabolism of cofactors and vitamins,” and “signal transduction and energy metabolism” were upregulated, while “membrane transport” and “nucleotide metabolism” were downregulated. The identified pathways in the three groups and their functions at the third classification level are shown in detail in Supplementary Figures S3–S5. We also observed that in C57BL/6 J mice, “cysteine and methionine metabolism,” “two-component system,” “oxidative phosphorylation,” “nitrogen metabolism,” “carbon fixation pathways,” and “sulfur metabolism” were upregulated, while “phosphotransferase system (PTS)” was downregulated, but in Mongolian gerbils and hamsters, “phosphotransferase system (PTS)” remained unchanged or showed trends opposite to those observed in C57BL/6 J mice.

## 4 Discussion

Even though there have been reports of animal models of CDI, significant differences among host species in terms of susceptibility to CDI are apparent, and host responses to CDI vary greatly depending on the animal species (Keel and Songer, 2006). This study is the first to report that Mongolian gerbils can be used as a model for CDI, to comparatively analyze gut microbiome among C57BL/6 J mice, Mongolian gerbils, and hamsters. Notably, the three rodent models presented different symptoms, degrees of intestinal histopathology, and changes in gut microbiome after *C. difficile* challenge. The hamsters challenged with *C. difficile* exhibited pathological changes, including explosive intestinal bleeding, and quickly died owing to severe enterocolitis. This result is consistent with those obtained in previous studies (Abutaleb and Seleem, 2021; Singh et al., 2022). C57BL/6 J mice did not manifest diarrhea, and furthermore, the pathological changes in their intestine were not significant. This finding is inconsistent with the previously reported findings (Chen et al., 2008; Wang et al., 2022). Histopathology in Mongolian gerbil is a typical histologic feature of CDI. Changes in their gut microbiome might be the main factor, leading to diverse response after *C. difficile* challenge.

An increasing number of studies have suggested that changes in gut microbiome composition play an important role in host health status (Zorio et al., 2019; Su et al., 2022). It has also been shown that a highly dense and diverse intestinal microbiome favors CDI prevention. Individuals colonized by *C. difficile* may not progress directly to symptomatic CDI, depending on the intestinal microenvironment, host immunity, and pathogen-related factors (AbdelKhalek and Narayanan, 2022; Reasoner et al., 2023). Furthermore, patients with CDI generally exhibit a significant loss of microbiota diversity, characterized primarily by the relative abundances of decreased Bacteroides, Firmicutes, *Lachnospiraceae*, and *Ruminococcaceae*, accompanied by an increase in the relative abundance of Proteobacteria, *Lactobacilliaceae, Enterococcaceae*, and *Streptococcaceae* (Milani et al., 2016; Gonzales-Luna et al., 2023). Additionally, *Enterococcus*, *Lactobacillus*, *Escherichia coli*, and *Akkermansia* are enriched in patients with CDI, and this enrichment is associated with various metabolic processes (Pérez-Cobas et al., 2014; Vakili et al., 2020a). In our studies, the gut microbiome of Mongolian gerbils after CDI was more similar to that of patients, as shown in decreased relative abundances of Bacteroides, *Lachnospiraceae*, and *Ruminococcaceae*, with corresponding increases in the relative abundances of Proteobacteria, *Lactobacilliaceae*, and *Akkermansia*. Thus, Mongolian gerbils showed different CDI symptoms than mice and hamasters and might show a similar trend with human in gut microbiota changes after *C. difficile* challenge. *Enterobacteriaceae* (Proteobacteria) are enriched in both animals and patients with CDI (Reeves et al., 2011; Schubert et al., 2014). The microbiota in rCDI patients is dominated by *Enterobacteriaceae* (Collins and Auchtung, 2017). *Enterobacteriaceae* are capable of thriving in the inflamed intestine and perpetuating continued inflammation (Bäumler and Sperandio, 2016). Thus, *Enterobacteriaceae* may have growth advantages in the gut environment of patients with CDI. Similar observations were made in this study; *Enterobacteriaceae* were enriched in Mongolian gerbils and hamsters challenged with *C. difficile*, and their overgrowth destroyed intestinal permeability and hence worsened colitis. The relative abundance of *Enterobacteriaceae* was lower in C57BL/6 J mice challenged with *C. difficile* than in challenged Mongolian gerbils and hamsters, and this animal showed no obvious intestinal tissue damage.

Some studies have shown that species of the family *Lactobacillaceae* can prevent the growth of pathogens (González-Lozano et al., 2022; Walter and O’toole, 2023). However, *Lactobacillaceae* was dominant in CDI samples from both humans and mice (Rea et al., 2011; Antharam et al., 2013). In this study, after challenge with *C. difficile*, we observed a significant decrease in the relative abundance of the *Lactobacillaceae* family in C57BL/6 J mice, while the *Lachnospiraceae* family became the most abundant Firmicutes. Contrasting observations were made for Mongolian gerbils and hamsters. These findings may be attributed to species of the family *Lactobacillaceae*, metabolizing hexoses via homofermentative or heterofermentative metabolism. KEGG functional enrichment analysis further revealed that PTS was decreased in C57BL/6 J mice but increased in Mongolian gerbils and hamsters. Reportedly, heterofermentative species of the family *Lactobacillaceae* harbor fewer PTS than homofermentative species (Zheng et al., 2015). Additionally, homofermentative species of the family *Lactobacillaceae* robustly acidify and inhibit the growth of *Lachnospiraceae* and *Muribaculaceae* (Lagkouvardos et al., 2019; Brownlie et al., 2022). A decrease in the abundance of *Lachnospiraceae*, resulting in low butyric acid production, may be associated with the development of ulcerative colitis (Sasaki et al., 2019). Additionally, *Muribaculaceae* can produce propionate as the main fermentation product, which, reportedly, is associated with gut health and an extended lifespan in mice (Smith et al., 2021). Furthermore, *Bacteroides* can degrade carbohydrates to form short-chain fatty acids (SCFAs), such as acetate, propionate, and butyrate (Koh et al., 2016). Notably, SCFAs can inhibit intestinal inflammation by upregulating G protein-coupled receptor 43 (GPR43), which facilitates the effects of hepatic portal pulsatile inflammatory factors (Maslowski et al., 2009). A decrease in the relative abundance of *Bacteroidetes* is associated with irritable bowel syndrome (Rajilić-Stojanović et al., 2011). Therefore, it is possible that, following challenge with *C. difficile* in Mongolian gerbils and hamsters, homofermentative species of the family *Lactobacillaceae* produce higher quantities of acid to inhibit the growth of *Lachnospiraceae* and *Muribaculaceae*, and the resulting low level of SCFAs may trigger intestinal pathology. However, in C57BL/6 J mice, although the abundance of *Muribaculaceae* was reduced, *Lachnospiraceae* and *Bacteroides* increased SCFA content to protect the intestine. Furthermore, in previous studies, C57BL/6 mice from different vendors had different levels of two different *Lactobacillus* spp. and had different responses to *H. pylori* infection, highlighting the importance of husbandry and the environment on the gastrointestinal microbiota profile (Rolig et al., 2013). This may also be the reason that mice are not sensitive to *C. difficile* challenge.

The changes in the relative abundance of *Akkermansia* were also interesting in three rodents challenged with *C. difficile*. In patients with CDI, an increased abundance of *Akkermansia* is associated with degraded mucin, which gives selective advantage to *C. difficile,* adhering to mucus (Hernández et al., 2018). Additionally, *Akkermansia* overrepresentation may reflect an epiphenomenon, leading to enteric mucosal inflammation with increased mucus production (Milani et al., 2016; Vakili et al., 2020b). An elevated abundance of *Akkermansia* may be a predictive marker for the presence of a CDI (Hernández et al., 2018). In this study, the abundance of *Akkermansia* also significantly increased in Mongolian gerbils challenged with *C. difficile*. *Akkermansia* was overrepresented in infected Mongolian gerbils, which was consistent with human CDI. However, we also observed increased *Akkermansia* levels in C57BL/6 J mice, and this genus was undetectable in hamsters challenged with *C. difficile*. *A. muciniphila,* as one of typical members in *Akkermansia,* found in the human gut, is a mucin-degrading bacterium that continuously remodels and refreshes the mucus layer, thereby creating a healthy environment for epithelial cells and maintaining intestinal barrier integrity (Everard et al., 2013). It has been reported that *A. muciniphila* could improve the abundance of *Akkermansia*, which alleviated intestinal inflammation reduced by CDI through ameliorating the intestinal barrier function and microbiota structure (Wu et al., 2022). This observation suggested that *Akkermansia* possibly played an important role in intestinal anti-inflammation in C57BL/6 J mice and hamsters challenged with *C. difficile*. Thus, the functions of *Akkermansia* might be diverse in three rodents. In Mongolian gerbils, the increased abundance of *Akkermansia* might be a potential predictive marker for CDI.

The results of this study also showed that some metabolic pathways were altered and, possibly, were related to CDI. Specifically, “glycan biosynthesis and metabolism” was upregulated in the three rodent groups, indicating that after challenge with *C. difficile,* each rodent model showed increased food utilization, which facilitated adaptation to pathogen infection. In CDI, sulfide, a direct precursor of both methionine and cysteine, plays an important role in sulfur metabolism (Dubois et al., 2016). It has also been shown that the accumulation of cysteine by-products can inhibit toxin production by *C. difficile* (Gu et al., 2017, 2018). Enhanced sulfur, cysteine, and methionine metabolism can also repress toxin production in *C. difficile*. Furthermore, the two-component signal transduction system (TCS) can sense and respond to environmental signals and its activation can inhibit sporulation in *C. difficile* (Kempher et al., 2022). In Mongolian gerbils and hamsters challenged with *C. difficile*, the reduced TCS could increase spore production, followed by subsequent CDI progression. Finally, our results indicated that the metabolism of cofactors and vitamins was upregulated in C57BL/6 J mice. Reportedly, vitamin B6 has pleiotropic functions in human health, e.g., it showed improved immune system activity (Hellmann and Mooney, 2010). This effect of vitamin B may also be the reason C57BL/6 J mice challenged with *C. difficile* did not manifest CDI symptoms, inconsistent with the results of the previous studies (Wu et al., 2022). Thus, the genetic backgrounds of mice of the same strain may not be completely consistent, owing to reproductive system differences between countries and regions. There were slight differences in feed formula composition, leading to different mouse intestinal flora; therefore, susceptibility to disease may be highly diverse.

This study had some limitations. First, the intestinal contents were used to infer changes in gut microbiome characteristics with a focus on just microbiome composition and function. Relevant data on metabolomics and transcriptomics might be helpful to further validate our findings. Second, we studied changes of gut microbiome, considering only the effect of *C. difficile* in this regard. Possibly, other factors, such as host response, affect the gut microbiome of the test animals. Thus, further investigations are required to investigate the role of other factors.

In this study, we established a Mongolian gerbil CDI model for the first time. The changes in the gut microbiome during challenge reflected the important role of the gut microbial community in the three rodent models analyzed. After *C. difficile* challenge, the three rodents showed a significant loss of microbiota diversity. The high abundance of species of the family *Lactobacillaceae* led to the production of higher quantities of acids to inhibit the growth of *Lachnospiraceae* and *Muribaculaceae*, leading to decreases in the levels of SCFAs and triggering intestinal pathology. Moreover, a decrease in the relative abundance of Bacteroidetes and an increase in that of Proteobacteria could also disrupt intestinal permeability and worsen colitis. Therefore, these phyla may be used as potential disease biomarkers or drug targets in the future. In addition, the increase in the *Akkermansia* population in both C57BL/6 J mice and Mongolian gerbils might result in different outcomes. Taken together, changes in gut microbiome constituted a key factor responsible for inducing the symptoms observed in the three rodent models following *C. difficile* challenge.

## Data availability statement

The original contributions presented in the study are publicly available. This data can be found at: https://www.ncbi.nlm.nih.gov/bioproject/; PRJNA1014570.

## Ethics statement

The animal study was approved by Laboratory Animal Center, Hangzhou Medical College. The study was conducted in accordance with the local legislation and institutional requirements.

## Author contributions

SW: Writing – original draft, Conceptualization, Data curation, Investigation, Methodology, Writing – review & editing. PY: Investigation, Writing – review & editing, Data curation, Methodology, Validation. QS: Writing – review & editing, Data curation, Methodology, Validation. HH: Methodology, Writing – review & editing, Formal analysis, Investigation, Software. LC: Writing – review & editing, Data curation, Formal analysis, Software, Validation. ZW: Software, Writing – review & editing, Data curation, Formal analysis, Validation. SL: Writing – review & editing, Methodology, Data curation, Investigation, Supervision. XS: Formal analysis, Writing – review & editing, Validation, Methodology, Software. YL: Writing – review & editing, Formal analysis, Data curation, Validation, Writing – original draft. YW: Resources, Writing – review & editing, Formal analysis, Software. DJ: Writing – original draft, Conceptualization, Funding acquisition, Investigation, Methodology, Supervision, Writing – review & editing, Resources. YC: Conceptualization, Data curation, Formal analysis, Funding acquisition, Investigation, Methodology, Project administration, Software, Supervision, Validation, Writing – original draft, Writing – review & editing. LZ: Data curation, Investigation, Writing – review & editing. FJ: Data curation, Investigation, Writing – review & editing.

## Footnotes

^1^ http://www.arb-silva.de/

^2^ http://drive5.com/usearch/manual/uclust_algo.html

^3^ https://mothur.org/

^4^ http://tax4fun.gobics.de
