# Supplementary figures and images for: Gut microbiome changes in mouse, Mongolian gerbil, and hamster models following Clostridioides difficile challenge
Source: Front Microbiol. 2024 Apr 4;15:1368194. doi: 10.3389/fmicb.2024.1368194 (PMC11024471; doi:10.3389/fmicb.2024.1368194)

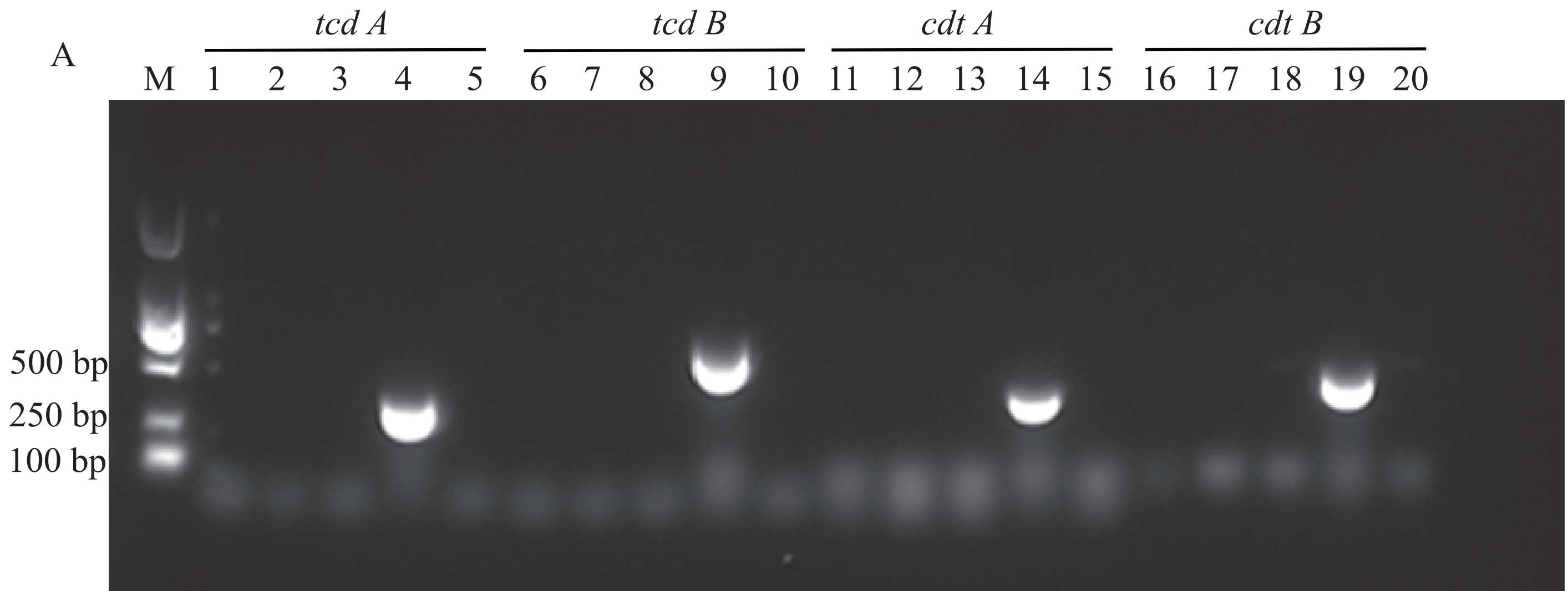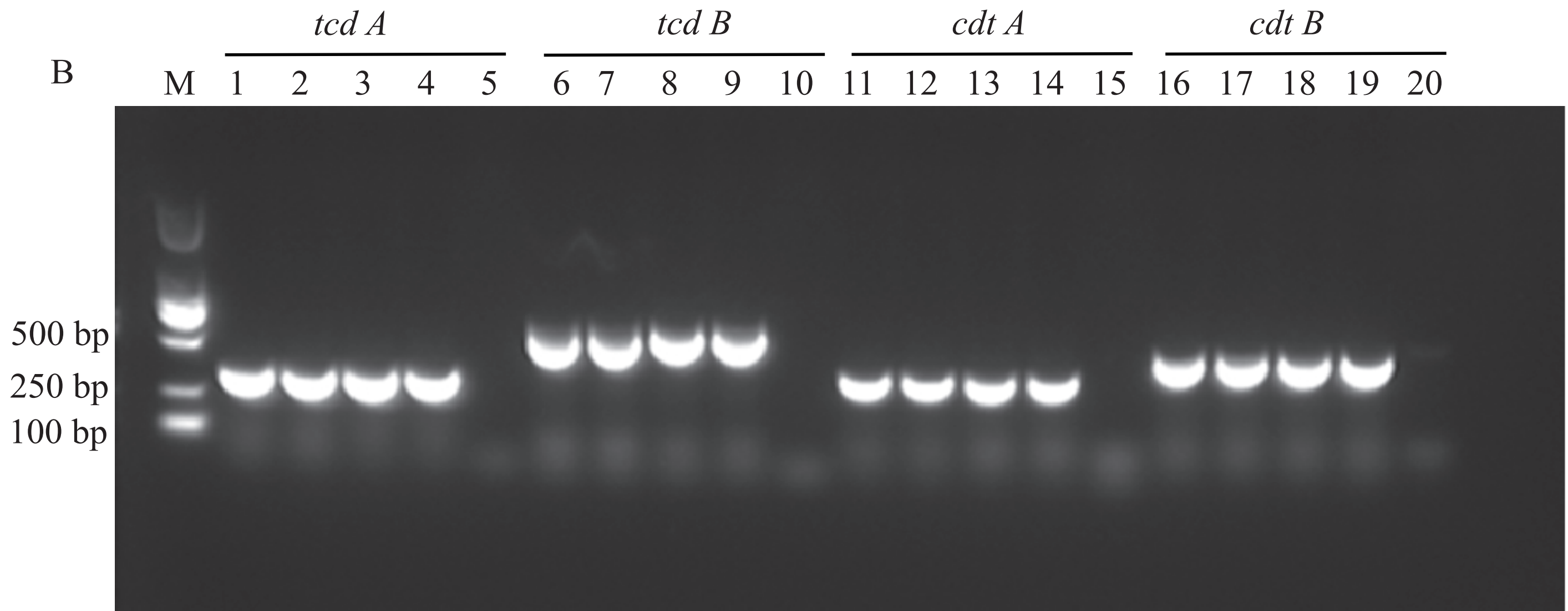

Supplement: Supplementary file 1 [file Data_Sheet_1.PDF]

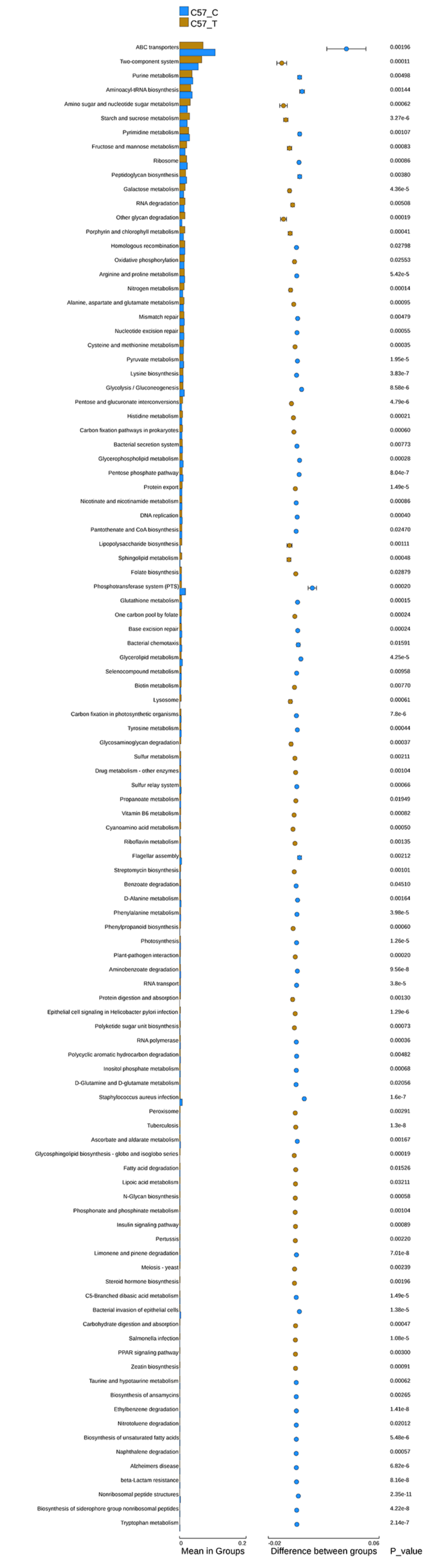

Supplement: Supplementary file 3 [file Data_Sheet_3.PDF]

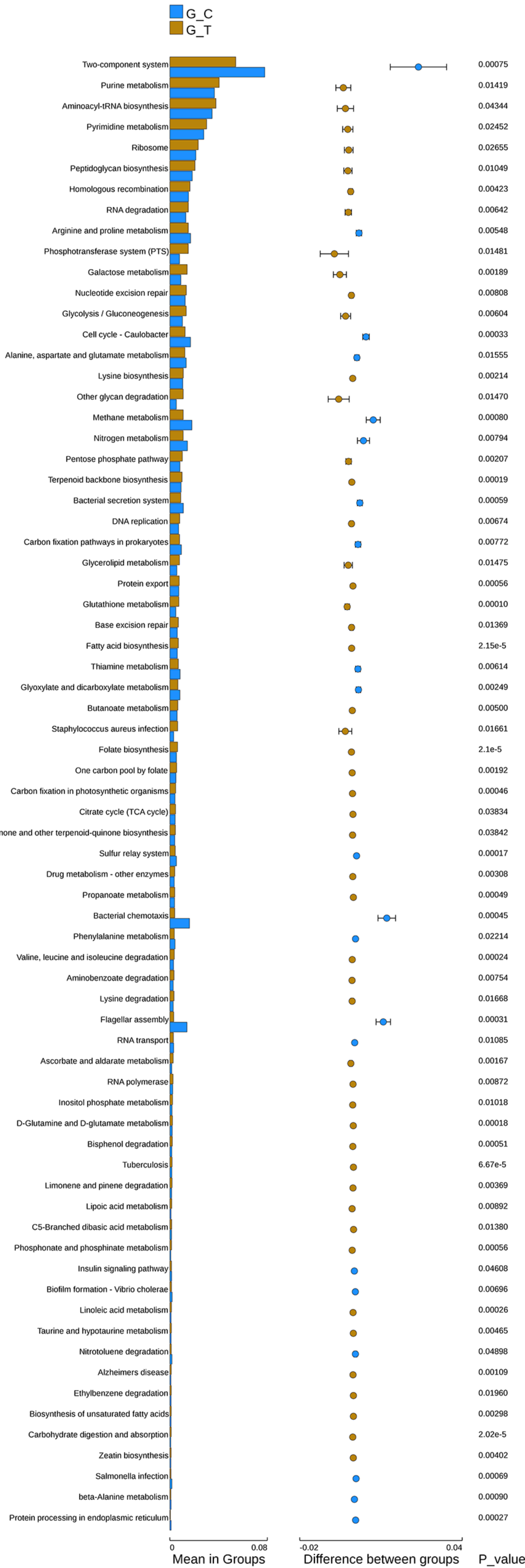

Supplement: Supplementary file 4 [file Data_Sheet_4.PDF]

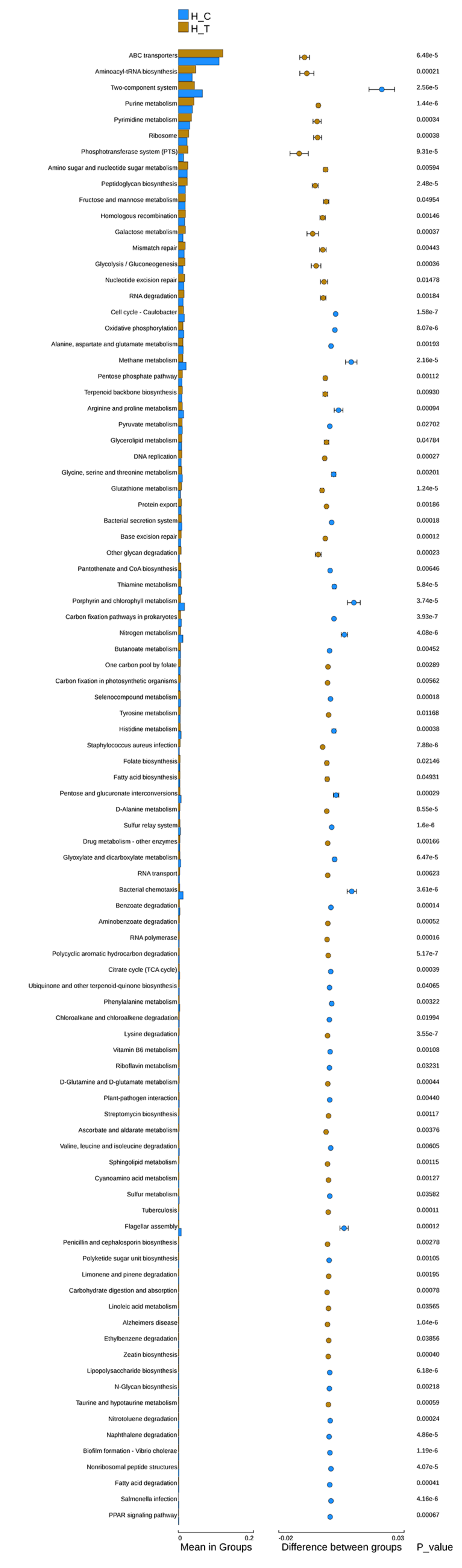

Supplement: Supplementary file 5 [file Data_Sheet_5.PDF]
