# Supplementary material for: Gut microbiome changes in mouse, Mongolian gerbil, and hamster models following Clostridioides difficile challenge
Source: Front Microbiol. 2024 Apr 4;15:1368194. doi: 10.3389/fmicb.2024.1368194 (PMC11024471; doi:10.3389/fmicb.2024.1368194)
Supplement: Supplementary file 2 [file Data_Sheet_2.PDF]

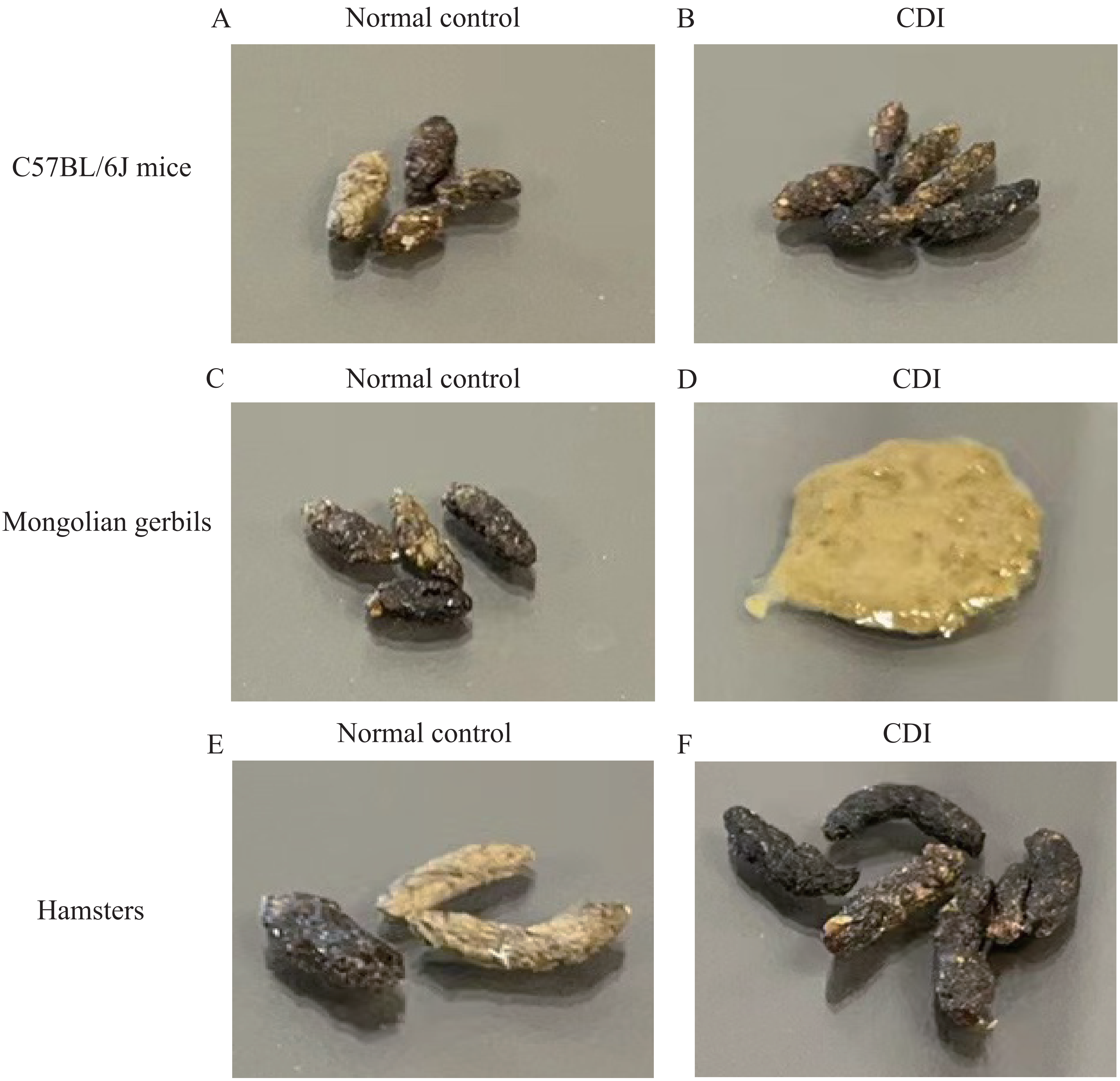
A
Normal control
B
CDI
C57BL/6J mice
C
Normal control
D
CDI
Mongolian gerbils
E
Normal control
F
CDI
Hamsters
